# Association of LOXL1 gene polymorphisms with exfoliation syndrome/glaucoma and primary open angle glaucoma in a Turkish population

**Published:** 2013-01-28

**Authors:** Burcu Kasım, Murat İrkeç, Mehmet Alikaşifoğlu, Mehmet Orhan, Mehmet Cem Mocan, Dilek Aktaş

**Affiliations:** 1 Department of Ophthalmology, Hacettepe University School of Medicine, Ankara, Turkey; 2 Department of Medical Genetics, Hacettepe University School of Medicine, Ankara, Turkey

## Abstract

**Purpose:**

To investigate the association of lysyl oxidase like 1 (*LOXL1*) variants with exfoliation syndrome (XFS), exfoliation glaucoma (XFG), and primary open angle glaucoma (POAG) in a Turkish population.

**Methods:**

Two *LOXL1* single nucleotide polymorphisms (SNPs), rs1048661 (R141L) and rs3825942 (G153D), were analyzed in 300 Turkish patients (100 patients with XFS, 100 patients with XFG, 100 patients with POAG) and 100 control subjects.

**Results:**

The T allele of rs1048661 was underrepresented in patients with XFS (odds ratio [OR]=0.334, 95% confidence interval [CI]: 0.198–0.564, p=2.54×10^−5^) and XFG (OR=0.366, 95% CI: 0.219–0.611, p=8.56×10^−5^) compared to the control subjects. None of the patients with XFS or XFG had the A allele of rs3825942, whereas 16% of the control subjects had that variant (OR=0.025, 95% CI: 0.003–0.188, p=3.69×10^−9^). No association was observed between the SNPs studied and POAG. By using logistic regression analysis, the effect of rs1048661 remained significant (p=8.45×10^−8^) after controlling for the effect of rs3825942, whereas rs3825942 was not significant with conditioning on rs1048661. Female gender was protective against the disease controlling with the effect of the two SNPs (OR=0.527, 95% CI: 0.358–0.776, p=0.001).

**Conclusions:**

The findings of the current study indicate that in a logistic regression analysis model the T allele of rs1048661 is the most important risk-modifying factor for the development of XFS and XFG. Our results also confirm in a Turkish population the findings of previous reports describing the association between *LOXL1* polymorphisms and XFS/XFG but not with POAG. The allele and genotype distribution in this cohort appear to be similar to those of Caucasians.

## Introduction

Exfoliation syndrome (XFS), first described by Lindberg in 1917, is an age-related disorder of the extracellular matrix, characterized by progressive accumulation of abnormal fibrillar material in many ocular and extraocular tissues [1]. Prevalence of XFS varies widely among populations and is highest in Scandinavian countries. The prevalence of XFS in Turkey is estimated to be around 10% to 15% [2].

XFS is the most common identifiable cause of secondary open angle glaucoma and associated with cataract and serious complications in cataract surgery. Exfoliation glaucoma (XFG) has a more severe prognosis, higher intraocular pressure (IOP) characteristics, and more severe optic nerve damage at the time of diagnosis and is more resistant to medical therapy than primary open angle glaucoma (POAG) [3].

In 2007, Thorleifsson et al. established a significant association between XFS and two non-synonymous single nucleotide polymorphisms (SNPs), rs1048661 (R141L) and rs3825942 (G153D), in exon 1 of the lysyl oxidase-like 1 (*LOXL1*) gene in Icelandic and Swedish individuals [4]. The association was confirmed in the United States [5,6], Australia [7], Austria [8], Finland [9], Germany [10], Italy [10], Poland [11], Pakistan [12], Saudi Arabia [13] and India [14]. However, in Chinese [15,16], Japanese [17-19] and Korean [20] populations, the risk allele was inverse for the rs1048661 SNP in contrast to Caucasians [5-13]. The purpose of this study was to investigate the exonic *LOXL1* SNPs, rs1048661 and rs3825942, variants in a Turkish population with XFS, XFG, and POAG.

## Methods

### Subjects

One hundred patients with XFS (44 males, 56 females), 100 patients with XFG (52 males, 48 females), 100 patients with POAG (46 males, 54 females) and 100 healthy controls (33 males, 67 females) evaluated at Hacettepe University School of Medicine, Department of Ophthalmology, Ankara, Turkey between May 2011 and May 2012 were recruited into this study. The mean age at blood-sampling was 70.8±8.8 years for XFS group, 72.5±8.3 years for XFG group, 67.7±9.3 years for POAG group and 66±5.7 years for control subjects. Informed consent was obtained from all participants. The study was approved by the ethics review board of the Medical School of Hacettepe University and adhered to the tenets of the Declaration of Helsinki. All subjects and controls underwent a comprehensive ocular examination, including visual acuity, slit-lamp examination, Goldmann applanation tonometry, optic disc examination, and measurement of central corneal thickness.

XFS was identified when the presence of exfoliation material was noted on the lens capsule, iris, or corneal endothelium with repeated in-office IOP measurements <21 mmHg and no clinical evidence of glaucomatous optic neuropathy in either eye. Pupillary dilation was performed on all subjects to determine the presence of exfoliation material on the anterior lens capsule. XFG was diagnosed if the patient had the criteria for XFS and the presence of glaucomatous optic disc cupping, visual field loss, and IOP ≥21 mmHg or controlled IOP on antiglaucomatous treatment in at least one eye. POAG was diagnosed in the presence of >1 IOP reading ≥21 mmHg, typical glaucomatous optic disc cupping with compatible visual field defects, and no sign of exfoliation material on the lens capsule, iris, or corneal endothelium in either eye. Control subjects were individuals older than >55 years of age without evidence of exfoliation deposits on anterior segment structures, who had repeated IOP readings of <21 mmHg and normal-appearing optic discs.

### Molecular analysis

After peripheral blood samples (5–10 ml) were collected from each subject, DNA was extracted with ammonium acetate precipitation [21]. SNPs rs1048661 (R141L) and rs3825942 (G153D) in exon 1 of *LOXL1* were amplified with predesigned primers and confirmed with polyacrylamide gel electrophoresis. Samples were incubated for 16 h with the restriction enzymes SmaI for R141L and HinfI for G153D at 25 °C and 37 °C, respectively. The genotyping was made by an investigator who was masked to the phenotypes. For rs1048661, after digestion with SmaI, the amplicon of 464 bp cleaved into fragments of 201 bp, 189 bp, and 74 bp genotyped as GG (wild-type) and 390 bp and 74 bp genotyped as TT (homozygous variant) and GT (heterozygous variant), which exhibits all the fragments (390 bp, 201 bp, 189 bp, and 74 bp). For rs3825942, after digestion with HinfI, the PCR amplicon that retained an intact fragment of 464 bp was genotyped as GG (wild-type), the variants that had a restriction site for the enzyme cleaved into fragments of 311 bp and 153 bp were genotyped as AA (homozygous variant), and the variants that had all fragments (464 bp, 311 bp, and 153 bp) were genotyped as GA (heterozygous variant). In all groups, the genotyping results were revalidated.

### Statistical analysis

Hardy–Weinberg equilibrium was tested for all case and control samples. The significance between the allele and genotype frequencies was assessed with chi-square analysis (Pearson chi-square test and Fisher’s exact test) using SPSS version 16.0 (IBM, Armonk, NY). For the alleles of the SNPs, the odds ratios, p values, and 95% confidence intervals were calculated. p<0.05 was considered statistically significant.

## Results

One hundred patients with XFS, 100 patients with XFG, 100 patients with POAG, and 100 control subjects were enrolled in this study. The mean age at blood-sampling was 70.8±8.8 years for the XFS group, 72.5±8.3 years for the XFG group, 67.7±9.3 years for the POAG group, and 66±5.7 years for the control subjects.

The genotype distribution of all SNPs conformed to Hardy–Weinberg equilibrium. Both *LOXL1* SNPs were significantly associated with XFS and XFG (Table 1 and Table 2). The T allele of rs1048661 was underrepresented in patients with XFS (OR=0.334, 95% CI: 0.198–0.564, p=2.54×10^−5^) and XFG (OR=0.366, 95% CI: 0.219–0.611, p=8.56×10^−5^) when compared to control subjects (Table 1 and Table 2). All patients (n=200) in the XFS and XFG groups had the G allele for rs3825942, which was detected more frequently than in the control subjects (n=168; Table 1 and Table 2), indicating that the A allele of rs3825942 is the most significant modifying factor for XFS with an OR of 0.025. There was no statistically significant difference between subjects with XFS and XFG for both SNP frequencies (p=0.762). The genotype frequencies of both SNPs in patients with XFS and XFG were also statistically different from those for the control subjects. For rs1048661, the TT and GT genotypes and, for rs3825942, the AA and GA genotypes were underrepresented in patients in the XFS and XFG groups when compared to control subjects (Table 1 and Table 2).

**Table 1:** Allele and genotype frequencies of rs1048661 and rs3825942 in exfoliation syndrome group and control subjects.

SNP	Control (n=100)	XFS (n=100)	OR (95% CI)	χ2	p
rs1048661 Allele					
T	58	24	0.334 (0.198–0.564)	17.733	2.54×10^–5^
G	142	176			
Genotype					
TT	10	1	0.068 (0.008–0.544)	16.475	2.64×10^–4^
GT	38	22			
GG	52	77			
rs3825942 Allele					
A	32	0	0.025 (0.003–0.188)	34.783	3.69×10^–9^
G	168	200			
Genotype					
AA	3	0	0.178 (0.020–1.628)	39.856	3.45×10^–10^
GA	26	0			
GG	71	100			

XFS: Exfoliation syndrome; OR: Odds ratio. Odds ratios were derived from the comparison of homozygous variant with common variant (TT versus GG at rs1048661, AA versus GG at rs3825942).

**Table 2:** Allele and genotype frequencies of rs1048661 and rs3825942 in exfoliation glaucoma group and control subjects.

SNP	Control (n=100)	XFG (n=100)	OR (95% CI)	χ2	p
rs1048661 Allele					
T	58	26	0.366 (0.219–0.611)	15.431	8.56×10^–5^
G	142	174			
Genotype					
TT	10	0	0.064 (0.08–0.513)	16.091	3.20×10^–4^
GT	38	26			
GG	52	74			
rs3825942 Allele					
A	32	0	0.025 (0.003–0.188)	34.783	3.69×10^–9^
G	168	200			
Genotype					
AA	3	0	0.178 (0.020–1.628)	39.856	3.45×10^–10^
GA	26	0			
GG	71	100			

XFG: Exfoliation glaucoma; OR: Odds ratio. Odds ratios were derived from the comparison of homozygous variant with common variant (TT versus GG at rs1048661, AA versus GG at rs3825942).

The effect of the two SNPs studied in XFS and XFG was determined by using logistic regression analysis. The effect of rs1048661 remained significant (p=8.45×10^−7^) after controlling for the effect of rs3825942. rs3825942 was not significant with conditioning on rs1048661 (p=0.997). By using logistic regression analysis, female gender was found to be protective against the disease controlling with the effect of the two SNPs (OR=0.527, 95% CI: 0.358–0.776, p=0.001).

No statistically significant differences in the allele and genotype frequencies of rs1048661 and rs3825942 were found between the patients in the POAG group and the control subjects (p=0.912 and p=0.198, respectively; Table 3).

**Table 3:** Allele and genotype frequencies of rs1048661 and rs3825942 in primary open angle glaucoma group and control subjects.

SNP	Control (n=100)	PAAG (n=100)	OR (95% CI)	χ2	p
rs1048661 Allele					
T	58	57	0.976 (0.633–1.505)	0.012	0.912
G	142	143			
Genotype					
TT	10	7	0.728 (0.257–2.062)	0.877	0.645
GT	38	43			
GG	52	50			
rs3825942 Allele					
A	32	42	1.370 (0.830–2.280)	1.472	0.225
G	168	158			
Genotype					
AA	3	6	2.219 (0.533–9.239)	1.618	0.476
GA	26	30			
GG	71	64			

POAG: Primary open angle glaucoma; OR: Odds ratio. Odds ratios were derived from the comparison of homozygous variant with common variant (TT versus GG at rs1048661, AA versus GG at rs3825942).

## Discussion

In this study, our results confirmed the association of two coding *LOXL1* SNPs with XFS and XFG for the first time in a Turkish population. Our findings appear to be similar to those in Caucasians [4-14] and differ from Chinese [15,16], Japanese [17-19], Korean [20], and South African [22,23] populations (Table 4). The G allele was detected more frequently in the XFS and XFG groups for both SNPs compared to the control group. Our findings suggest that the T allele for rs1048661 and the A allele for rs3825942 appear to be protective for XFS and XFG. The A allele of rs3825942 was the greatest protective factor with an OR of 0.025 for XFS. However, following logistic regression analysis, rs3825942 was no longer significant for the development of XFS or XFG after controlling for the effect of rs1048661 while rs1048661 remained significant. Thus, the findings of the current study suggest that the effect of rs1048661 on XFS and XFG is independent of rs3825942. In contrast, the AA genotype of rs3825942 conferred the greatest risk for XFS in the South African population [23], a finding that contradicts the assumption that the G allele and the GG genotype are linked to the development of XFS. No statistically significant differences were shown in allele frequencies for both SNPs between XFS and XFG. Our findings suggest that the common variants of *LOXL1* SNPs are responsible for disease onset but not IOP elevation.

**Table 4:** Summary of allele frequencies of rs1048661 and rs3825942 as risk modifying factors for exfoliation syndrome/exfoliation glaucoma reported in various populations.

Studied population	Case	Control	Significance	Case	Control	Significance
Austria (Mossböck et al.) [8]	0.159	0.329	2.55×10^–7^	0.006	0.183	5.76×10^–15^
China (Chen et al.) [15]	0.89	0.52	6.95×10^–11^	0	0.1	8.00×10^–4^
China (Lee et al.) [16]	0.476	0.556	0.142	0.008	0.082	0.0018
Finland (Lemmelä et al.) [7]	0.175	0.317	2.65×10^–5^	0.032	0.177	2.24×10^–8^
Germany (Pasutto et al.) [10]	0.182	0.356	4.32×10^–16^	0.049	0.143	1.21×10^–11^
Iceland (Thorleifsson et al.) [4]	0.219	0.349	1.80×10^–6^	0.016	0.153	4.10×10^–9^
Italy (Pasutto et al.) [10]	0.175	0.307	0.0009	0	0.179	1.66×10^–18^
Japan (Fuse et al.) [17]	0.964	0.507	7.70×10^–18^	0	0.123	4.10×10^–4^
Japan (Mabuchi et al.) [18]	0.006	0.45	<0.0001	0.006	0.147	<0.0001
Korea (Sagong et al.) [20]	0.93	0.64	5.74×10^–12^	0.02	0.11	0.0003
Poland (Malukiewicz et al.) [11]	0.097	0.2	0.09	0	0.133	0.005
Pakistan (Micheal et al.) [12]	0.148	0.342	1.00×10^–7^	0.027	0.161	1.00×10^–7^
Saudi Arabia (Abu-Amero et al.) [13]	0.124	0.238	0.0056	0.032	0.183	5.00×10^–6^
South Africa (Williams et al.) [23]	0.01	0.19	1.70×10^–5^	0.87	0.38	5.20×10^–13^
South Africa (Rautenbach et al.) [21]	0	0.117	0.00106	0.86	0.383	0.00582
Sweden (Thorleifsson et al.) [4]	0.166	0.318	2.70×10^–7^	0.005	0.121	9.10×10^–14^
USA (Challa et al.) [6]	0.213	0.335	0.0222	0.061	0.156	0.0194
USA (Aragon-Martin et al.) [5]	0.157	0.297	7.74×10^–19^	0.041	0.202	3.10×10^–17^
Turkey-The current study	0.125	0.29	7.08×10^–7^	0	0.16	5.8×10^–16^

The *LOXL1* gene is a member of the lysyl oxidase family. A functional complex of *LOXL1* with elastin fibers is thought to be essential for elastogenesis [24]. In a recent study, *LOXL1* was shown to be the major lysyl oxidase isoform in normal lamina cribrosa [25]. In the late stages of XFS, significant downregulation of *LOXL1* messenger RNA (mRNA) and elastic protein levels was demonstrated [25]. This alteration in *LOXL1* regulation and elastogenesis in XFS possibly leads to the vulnerability to optic nerve damage even in lower levels of IOP [25]. In the original study by Thorleifsson et al., the expression of *LOXL1* mRNA was analyzed in the presence of the reported variants [4]. In that report, the G allele of rs1048661 was associated with a reduction in *LOXL1* mRNA by 7.7% in adipose tissue, while the variants of rs3825942 had no effect. Similar findings were shown in intraocular tissues in another study by Schlötzer-Schrehardt et al. [26]. In our study, the effect of rs3825942 on the disease was shown to be dependent on rs1048661. These findings suggest that the variants of rs3825942 may have functional effects on *LOXL1*, such as substrate targeting or enzyme activity when the common variant of rs1048661 exists [4-13,15-20].

No significant association was found between *LOXL1* variants and POAG in previous studies [9,18,21,27-29]. This finding was verified in our study population. This finding implies that *LOXL1* SNPs are associated with the pathogenesis of exfoliation syndrome but not IOP elevation or the glaucomatous process. The risk alleles of both SNPs were also commonly detected in control subjects (71% for rs1048661 and 84% for rs3825942), implying that the presence of high-risk *LOXL1* polymorphisms is not sufficient to give rise to XFS; other copathogenetic mechanisms may be necessary for the disease to manifest.

In the initial genome-wide association study undertaken by Thorleifsson et al., an intronic sequence variant rs2165241 in the *LOXL1* gene was strongly associated with the phenotypic expression of XFS [4]. However, the association of the intronic sequence variant was not significant after adjusting for non-synonymous SNPs simultaneously. In more recent reports as well as in the current study, only the two exonic SNPs rs1048661 and rs3825942 were evaluated for their possible association with XFS [14,23,30].

In conclusion, our findings reveal that the two exonic sequence variants residing on the *LOXL1* gene, rs1048661 and rs3825942, were associated with XFS and XFG, but not with POAG. To the best of our knowledge, this study is the first to identify the *LOXL1* gene variants and to reveal the T allele of rs1048661 as the most significant risk-modifying factor for XFS and XFG in Turkish individuals. Our findings may have potential value in early identification of individuals at risk for developing XFS.
